# The association between the timing of initial hepatitis B vaccination and seropositivity in healthcare personnel

**DOI:** 10.1017/ice.2024.107

**Published:** 2024-11

**Authors:** Elizabeth H. Lees, M. Hassan Murad, Laura E. Breeher, Melanie D. Swift

**Affiliations:** Division of Public Health, Infectious Diseases, and Occupational Medicine, Mayo Clinic, Rochester, MN, USA

## Abstract

This study reports on the relationship between timing of initial hepatitis B virus (HBV) vaccine series and HBV antibody immunity in healthcare personnel (HCP) screened prior to employment. HCPs vaccinated as neonates were significantly more likely to have negative or indeterminate antibodies. An alternative screening approach is considered.

## Introduction

It is current practice to screen for hepatitis B virus (HBV) immunity as part of preplacement exams for healthcare personnel (HCP). Circulating HBV antibodies can wane over time, and as employees who were vaccinated at birth in the 1990s enter the workforce, employers face a new challenge in determining whether previously vaccinated employees are protected from HBV infection. Challenge doses of HBV vaccine are used to demonstrate immunity when initial antibody screening is negative or indeterminate, and most vaccinated individuals mount a robust anamnestic response to a single challenge dose of HBV vaccine, which suggests the presence of long-lasting cellular immunity.^1,2^

Current guidelines for screening HCP in the United States are to draw a HBsAb, and if negative, to administer one challenge dose of HBV vaccine followed 4–8 weeks later by another HBsAb test.^3,4^ If the post-challenge HBsAb remains negative, the HCP receives a repeat HBV vaccine series.^3,4^ The recommendations are the same for all HCPs regardless of individual factors that may predict a higher likelihood of a negative screen. A predictive model based on individual factors such as current age, time since vaccination, and/or age at vaccination could make this process more efficient. Our study aimed to examine the relationship between timing of a medical student’s initial HBV vaccine series and HBsAb screening results. Based on observations, we suggest a new approach to HBV preplacement screening.

## Methods

Data were collected from two cohorts of medical students. Preplacement HBV immunity screening was performed by collecting HBsAb. Students with a negative or indeterminate HBsAb were administered a challenge dose of HBV vaccine and retested 1–2 months later. If the HBsAb was not positive upon retesting, additional HBV vaccine dose(s) were administered to complete the second series. There were 108 students in the study group. Applying exclusion criteria left 85 students. Exclusion criteria included students who could not provide vaccine documentation, did not complete required serology testing, or declined research participation. Students with documentation of a positive HBsAb at any time following their initial vaccine series were also excluded since prior studies have shown that a positive HBsAb at any time serves as lifetime proof of immunity. This included students vaccinated as infants who had prior proof of positive HBsAb.

The laboratory upper and lower limits for HBsAb were defined: positive (HBsAb >12 mIU), negative (HBsAb <5 mIU), and indeterminate (HBsAb 5–12 mIU). A challenge dose response was also defined as HBsAb >12 mIU. For the purposes of data analysis, students with indeterminate HBsAb were counted with students who had negative HBsAb since both groups were administered an HBV challenge dose. The students were categorized according to age of first documented HBV vaccines. The neonate group included those who received initial HBV vaccine within the first 30 days of life. The pediatric group included those vaccinated between 31 days and 18 years of life. The adult group included students vaccinated after the age of 18 years.

SAS OnDemand was utilized for statistical modeling. Logistic regression was used to analyze the relationship between HBsAb, age at vaccination, sex, race, and medical comorbidities. An additional logistic regression model was used to independently assess the relationship between HBsAb status and years between the most recent HBV vaccine and HBsAb test. This was done to determine whether outcomes from the first model were more attributable to age group at time of initial vaccine series or due to time elapsed between the vaccine and the HBsAb test.

## Results

There were 35 students vaccinated in the neonate age group, 44 in the pediatric age group, and 6 students in the adult age group. Student characteristics are shown in Table 1. The group vaccinated as neonates resulted in the highest proportion of negative or indeterminate HBsAb at the time of screening (74.3%). The group vaccinated as pediatrics had 25% negative or indeterminate HBsAb antibody results, and those with adulthood vaccination had 16.7% negative or indeterminate results. Logistic regression was used to predict the odds of having a negative or indeterminate HBsAb after adjusting for timing of initial vaccine series, sex, race, and medical comorbidities. Compared to those vaccinated as adults, students vaccinated as neonates had significantly higher odds of negative or indeterminate HBsAb (13.8, 95% CI (1.3, 141.8) and *P* = 0.0007). The odds for the pediatric vaccination group compared to those vaccinated as adults were not significant, nor were the odds significant for differences in race, sex, or medical comorbidities (Table 2).

**Table 1.** Baseline characteristics of medical student participants

Variable of interest	Variable subgroups	All (n)	Serum antibody status	
			Negative/Indeterminate	Positive
Sex	Male	40	14	26
	Female	45	24	21
Race	White	49	22	27
	Asian/Indian	18	8	10
	Black	7	3	4
	Other	8	3	5
Comorbidities	Nonautoimmune	34	15	19
	Autoimmune	7	3	4
	Immunosuppress. Meds	4	2	2
	None	40	18	22
Age Group (at time of vaccination)	Neonate	35	26	9
	Pediatric	44	11	33
	Adult	6	1	5

**Table 2.** Odds ratios for logistic regression model of serum antibody immunity adjusting for timing of initial vaccine series, sex, race, and comorbidities

Effect	Odds Ratio	95% Confidence Interval	*P*-value
Neonate age group	13.8	(1.34, 141.8)	0.0007
Pediatric age group	1.3	(0.1, 13.2)	0.1
Female	1.9	(0.7, 5.5)	0.2
Nonwhite	0.5	(0.2, 1.6)	0.2
Comorbidities	1.2	(0.4, 3.3)	0.7

For students with an initial negative or indeterminate HBsAb, 88.6% responded to challenge dose. The neonatal vaccination group had the largest percentage of challenge dose nonresponders (13%), followed by the pediatric vaccination group (9%). All students in the adult group with negative or indeterminate HBsAb had a positive response to challenge dose. Logistic regression did not demonstrate any significant relationships between predictor variables and response to challenge dose.

The mean number of years between completion of initial HBV vaccine series and obtaining HBsAb for the neonatal vaccine group was 23.9 years. The mean number of years for the pediatric group was 19.2, and the mean number for the adult group was 4.2 years. Logistic regression was used to predict the odds of having a negative or indeterminate HBsAb and number of years between last HBV vaccine and to HBsAb test. Models were tested for both the neonate and pediatric age groups. There was no significant association for the neonatal or pediatric vaccination age groups (OR 1.2, 95% CI (0.7, 2.2) and OR 1.1, 95% CI (1.0, 1.3) respectively).

## Discussion

This retrospective study demonstrated that timing of initial HBV vaccine series is a predictor of HBsAb status when performed as part of preemployment procedure. Students vaccinated as neonates (within the first 30 days of life) were significantly more likely to have negative or indeterminate HBsAb compared to their peers who were vaccinated later in childhood or as adults. This observation was independent of sex, race, or medical comorbidities. This relationship was also independent of the years between HBV vaccine and HBsAb testing, suggesting premature immune system response during the neonatal period may be the more likely cause of waning HBsAb than passage of time.

The results of our study indicate that individuals who were vaccinated in the neonatal period of infancy are at the highest risk of negative or indeterminate HBsAb at the time of preemployment screening. Given these results, employers might consider offering a preplacement challenge dose, followed in 4–8 weeks by a screening HBsAB, for individuals vaccinated as neonates without any prior proof of HBsAb. This approach would eliminate the need for initial serology testing that has a high chance of being negative or indeterminate for individuals vaccinated in early infancy.

Small sample size was a limitation of this study, and future studies would benefit from a larger sample with greater diversity. Another limitation was that the study design was a retrospective view of an existing program, and we were unable to perform additional assays to identify individuals whose positive HBsAb might have been accompanied by a robust IgM response to sAb, thus indicating newly acquired vaccine-mediated immunity. Finally, future studies are needed to assess the full cost-savings potential of a preplacement screening procedure that administers challenge doses to all individuals vaccinated as neonates without proof of a positive HBsAb versus standard procedure with a screening HBsAb.

## Supporting information

Lees et al. supplementary material Lees et al. supplementary material

## Supplementary material

The supplementary material for this article can be found at https://doi.org/10.1017/ice.2024.107.
