# Supplementary figures and images for: The association between the timing of initial hepatitis B vaccination and seropositivity in healthcare personnel
Source: Infect Control Hosp Epidemiol. 2024 Sep 16;45(11):1347–9. doi: 10.1017/ice.2024.107 (PMC11663467; doi:10.1017/ice.2024.107)

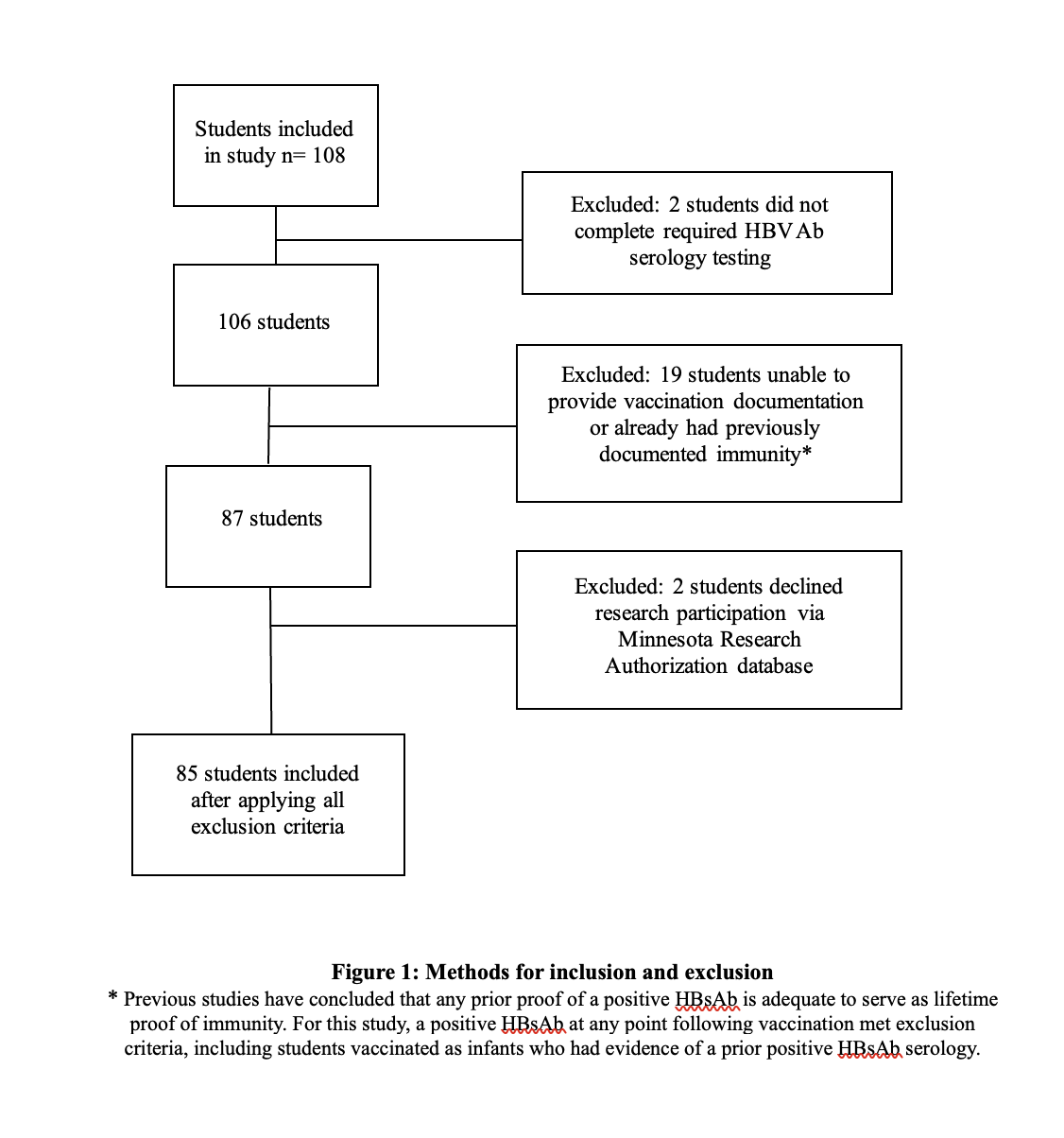

Supplement: Lees et al. supplementary material [file S0899823X24001077sup001.docx]
